# A structural and functional comparison of gap junction channels composed of connexins and innexins

**DOI:** 10.1002/dneu.22447

**Published:** 2016-11-24

**Authors:** I. Martha Skerrett, Jamal B. Williams

**Affiliations:** ^1^ Biology DepartmentSUNY Buffalo State, 1300 Elmwood AveBuffaloNew York14222

**Keywords:** gap junction, innexin, connexin, structure, function

## Abstract

Methods such as electron microscopy and electrophysiology led to the understanding that gap junctions were dense arrays of channels connecting the intracellular environments within almost all animal tissues. The characteristics of gap junctions were remarkably similar in preparations from phylogenetically diverse animals such as cnidarians and chordates. Although few studies directly compared them, minor differences were noted between gap junctions of vertebrates and invertebrates. For instance, a slightly wider gap was noted between cells of invertebrates and the spacing between invertebrate channels was generally greater. Connexins were identified as the structural component of vertebrate junctions in the 1980s and innexins as the structural component of pre‐chordate junctions in the 1990s. Despite a lack of similarity in gene sequence, connexins and innexins are remarkably similar. Innexins and connexins have the same membrane topology and form intercellular channels that play a variety of tissue‐ and temporally specific roles. Both protein types oligomerize to form large aqueous channels that allow the passage of ions and small metabolites and are regulated by factors such as pH, calcium, and voltage. Much more is currently known about the structure, function, and structure–function relationships of connexins. However, the innexin field is expanding. Greater knowledge of innexin channels will permit more detailed comparisons with their connexin‐based counterparts, and provide insight into the ubiquitous yet specific roles of gap junctions. © 2016 Wiley Periodicals, Inc. Develop Neurobiol 77: 522–547, 2017

## EARLY STRUCTURAL STUDIES OF GAP JUNCTIONS REVEAL MINOR DIFFERENCES BETWEEN GAP JUNCTIONS OF VERTEBRATES AND INVERTEBRATES

**Figure 1:**
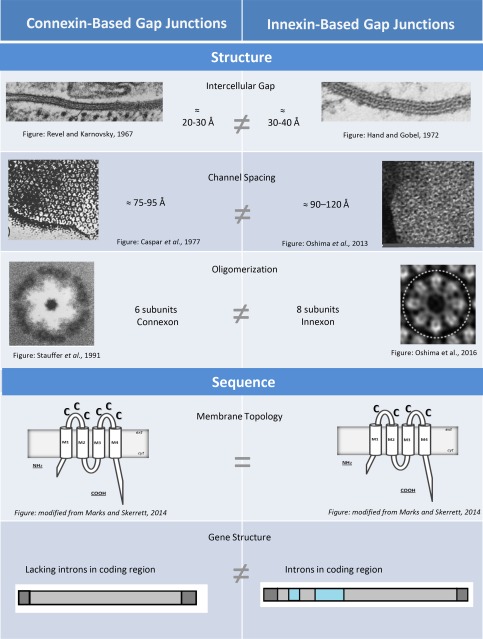
Comparison of gap junctions composed of connexins and innexins focusing on structure and sequence. Representative images are not adjusted to scale. Intercellular Gap: The gap between cells is slightly larger in invertebrate preparations. (Left) Section of mouse heart gap junction treated *en bloc* with lanthanum and stained with uranyl acetate. X 200,000. Intercellular gap ≈ 18 Å. Revel and Karnovsky, 1967. (Right) Section of a gap junction between muscle cells of Hydra treated *en bloc* with lanthanum and stained with lead citrate. X 144,000. Intercellular gap ≈ 30 Å. Hand and Gobel, 1972. Channel Spacing: Channels are spaced farther apart in invertebrate preparations. (Left) Electron micrograph of an isolated gap junction plaque from mouse liver. Center of connexons are marked. Caspar et al., 1977. (Right) Electron micrograph of gap junction plaque from Sf9 cells expressing *c. elegans* INX‐6 negatively stained with uranyl acetate. Oshima et al., 2013. Oligomerization: Connexin‐based channels are hexameric while innexin‐based channels are octameric. (Left) Sixfold rotationally filtered image of a connexon purified from rat liver (Stauffer et al., 1991). (Right) Projection map of a *c. elegans* INX‐6 deletion mutant expressed in Sf9 cells, solubilized, purified and negatively stained. Membrane Topology: The membrane topology of proteins that constitute gap junctions. Both connexins (left) and innexins (right) have four membrane‐spanning domains, two extracellular loops and cytoplasmic amino and carboxyl termini. Each of the two extracellular loop domains includes three conserved cysteines in connexins and two conserved cysteines in innexins. Gene Structure: Illustration summarizing gene structure of connexins (left) and innexins (right). Light grey denotes coding region bracketed by small sections of untranslated sequence (dark gray). Representative Introns are noted in cyan. Connexin genes do not contain introns within the coding region whereas innexin genes contain introns.

Gap junctions are dense arrays of hundreds or thousands of intercellular channels in regions where cell membranes are closely apposed. The earliest structural studies of gap junctions involved thin section electron microscopy (EM). In most cases thin sections were studied after tissues were treated with an electron‐opaque material involving lanthanum hydroxide or ruthenium red, which permeates the gap between cells providing contrast at regions of close apposition (Dewey and Barr, 1962; Robertson, 1963; Benedetti and Emmelot, 1965; Revel and Karnovsky, 1967). In the 1960s and 1970s, thin‐section EM was applied to junctions from a wide range of animal tissues including Mauthner cell club endings (Robertson, 1963), mouse heart and liver (Revel and Karnovsky, 1967), cockroach epidermis (Hagopian, 1970), smooth muscle cells of sheep (Uehara and Burnstock, 1970), Daphnia epithelium (Hudspeth and Revel, 1971), several types of tissue in Hydra (Hand and Gobel, 1972), and lateral giant fibers of crayfish (Peracchia, 1973). These studies produced images of a seven‐layered (septilaminar) structure about 150–190 Å wide present in nearly all animal tissue types (Gilula, 1978; Leitch, 1992). The gap between opposed membranes ranged from 20 to 60 Å (Table 1). While these early studies focused primarily on the prevalence of gap junctions in animal tissue, differences in gap width were apparent in close comparisons of vertebrate and invertebrate junctions (Fig. 1, Intercellular Gap). Vertebrate junctions tend to have a narrower gap, in the range of 20–30 Å (Revel and Karnovsky, 1967; Uehara and Burnstock, 1970) compared to 30–40 Å gap for invertebrate tissue (Hand and Gobel, 1972; Peracchia, 1973).

**Table 1:** Common Morphological Features of Gap Junctions as Noted for a Few Vertebrate and Invertebrate Preparations

Feature	Vertebrate (Å)	Invertebrate (Å)
Intermembrane spacing (GAP)	20‐30 (Revel and Karnovsky, 1963) 25‐30 (Uehara and Burnstock, 1970) 25‐35 (Sosinsky et al., 1988)	30 (Hand and Gobel, 1972) 30‐40 (Peracchia, 1973) 30‐40 (Flower, 1977) 60 (Oshima et al., 2016)
Unit Cell (center to center distance between channels)	77–94 (Oshima et al., 2013). 81‐88 (Caspar et al., 1977) 90–95 (Revel and Karnovsky, 1963) 90 (Robertson, 1963)	100‐110 (Oshima et al., 2013) 125‐200 (Peracchia, 1973) 198 (Ohta et al., 2011) 90‐100 (Flower, 1971) 120 (Flower, 1977)
GJ thickness (end‐to‐end channel length)	150 (liver, Caspar et al., 1977) 155 (liver, Sosinsky et al., 1988) 155 (Cx26, Maeda et al., 2009) 174 (Cx26, Müller et al.,^2002^) 250 (Cx43, Yeager, 1998) 140–162 (Cx26, Oshima et al., 2013)	170 (arthropod, Peracchia, 1973) 184 (INX‐6, Oshima et al., 2013) 240 (INX‐6, Oshima et al., 2016

In some early studies, *en face* views of tracer‐free (Robertson, 1963) and tracer‐impregnated gap junctions (Benedetti and Emmelot, 1965; Goodenough and Revel, 1970) were also obtained in which distinct subunits appeared in a polygonal (often hexagonal) lattice. Within this lattice, center‐to‐center measurements of subunits revealed spacing of 90–100 Å (see Table 1, reviewed by Gilula, 1978).

### Freeze‐Fracture Analysis of Gap Junctions

By the 1970s freeze‐fracture methods were commonly applied to gap junctions Freeze‐fractured gap junction membranes contain two complementary fracture faces, a cytoplasmic face (p/face) and an extracellular face (e/face). Vertebrate gap junctions yield a particle‐embedded p/face containing the polygonal lattice of subunits and an e/face with the complementary set of pits or depressions (Gilula, 1978). Some of the first noted differences between vertebrate and invertebrate gap junctions were related to the appearance of these fracture faces. With the exception of preparations from mollusk (Flower, 1971), invertebrate gap junctions yielded pits or depressions on the p/face with subunits remaining embedded on the e/face. This is the reverse of what was observed in vertebrate junctions, and to reflect the difference, the terms A‐ and B‐ type junctions were coined for vertebrate and invertebrate junctions, respectively (Flower, 1977; Gilula, 1978). Since most studies of invertebrate junctions involved arthropod tissue, the “invertebrate” B‐type junctions originally referred specifically to gap junctions from the phylum Arthropoda (reviewed by Gilula, 1978). However, this was later expanded to include other phyla such as Coelenterata, Platyhelminthes, and Annelida (Flower, 1977).

In almost all preparations, gap junctions include hundreds or thousands of channels that appear as tightly packed recessions or pits (Flower, 1971; Peracchia, 1973; Leitch, 1992; Sosinsky, 1992). In clear images it is apparent that the channels are surrounded by protrusions suggestive of subunits arranged around a central pore (Peracchia, 1973; Leitch, 1992; Sosinsky, 1992). Consistent differences are apparent when the size and spacing of the pits are compared between vertebrate and invertebrate preparations. However, caution must be exercised in generalizing information related to size and spacing of channels from different preparations. Variations are evident within preparations, between preparations from the same organism, and between preparations from different organisms. Variation within the same preparation may represent different gating states of gap junction channels, while variation between preparations may represent procedural differences in preparing tissue for analysis, different gating states or different data interpretation methods. Overall gap junction channels appear larger and have a greater intermembrane spacing in invertebrate preparations (Fig. 1, Channel Spacing). Table 1 summarizes dimensions of individual channels and measurements of the “unit cell” in various preparations. The unit cell is a measure of the distance between adjacent channels in a hexagonal array and ranges from 77 to 95 Å in vertebrate preparations (Revel and Karnovsky, 1963; Robertson, 1963; Caspar et al., 1977; Larsen, 1977) and 100–200 Å for invertebrate preparations (Peracchia, 1973; Flower, 1977; Larsen, 1977; Leitch, 1992).

When combined with more recent studies involving methods such as cryo‐EM (Unger et al., 1997; Oshima et al., 2008; Oshima et al., 2016), electron tomography (Ohta et al., 2011), atomic force microscopy (AFM; Müller et al., 2002), and X‐ray diffraction (Caspar et al., 1977; Maeda et al., 2009; Bennett et al., 2016) there is clear evidence for structural differences between vertebrate and invertebrate junctions. Gap junctions of invertebrates appear to have a greater end‐to‐end length. Typical estimates of end‐to‐end length of invertebrate channels range from 170 to 184 Å (Peracchia, 1973; Leitch, 1992; Blagburn et al., 1999; Oshima et al., 2013) compared to 140–250 Å for vertebrate preparations (Sosinsky et al., 1988; Yeager, 1998; Müller et al., 2002; Maeda et al., 2009). These ranges are likely due to the variation in size between subunits. In invertebrates the larger innexin subunits appear to result in less variation, while the vertebrate connexins have a broad distinction in mass and length attributed to the differences in carboxyl terminal tail length. For instance, Cx26 has the shortest cytoplasmic C‐terminus which correlates with the smallest end‐to‐end measurement of vertebrate junction thickness (Sosinsky et al., 1988; Müller et al., 2002). Under similar conditions, the width of channels composed of Cx26 and Cx43 are 140 Å and 162 Å, respectively (Oshima et al., 2013). Gap junction width (end‐to‐end channel length) is also calcium‐dependent, with higher calcium concentrations inducing thicker preparations presumably due to ordering of cytoplasmic regions (Müller et al., 2002).

While there is considerable evidence for a distinction between vertebrate and invertebrate gap junctions in terms of gap width and channel size/spacing, until very recently there appeared to be little evidence that the oligomeric status of channels composed of connexins and innexins differed. In cases where subunits could be resolved as single protrusions arranged a central pore, six subunits were noted in vertebrate (Zampighi and Unwin, 1979; Baker et al., 1983; Müller et al., 2002; Sosinsky and Nicholson, 2005) and invertebrate (Peracchia, 1973; Ohta et al., 2011; Oshima et al., 2013) preparations, consistent with hexameric connexons channels (Sosinsky and Nicholson, 2005). The hexameric nature of connexons has been confirmed in several models of Cx43 and Cx26 (Unger et al., 1999; Oshima et al., 2008; Maeda et al., 2009; Bennett et al., 2016) with a similar arrangement cautiously predicted for invertebrate channels (Peracchia, 1973; Oshima et al., 2013). However, when studied by cryo‐EM at 10 Å resolution, reconstituted gap junction channels composed of *c. elegans* INX‐6 revealed individual innexons involving eight subunits (Oshima et al., 2016). The complete INX‐6 channel was described as hexadecameric (consisting of 16 subunits). While further studies are required to confirm a hexadecameric structure for other invertebrate gap junction channels, the results are consistent with many years of accumulated work indicating that invertebrate channels are larger and more widely spaced than their invertebrate counterparts (Fig. 1, Oligomerization).

## IDENTIFICATION OF CONNEXINS AND INNEXINS AS THE GAP JUNCTION PROTEINS OF CHORDATES AND PRE‐CHORDATES, RESPECTIVELY

### Connexins

Soon after gap junctions were identified as structural and functional components of intercellular junctions, attempts were made to discover their protein make‐up. The earliest studies involved proteolysis and identification of protein fragments (Goodenough and Stoeckenius, 1972; Goodenough, 1974) with subsequent studies identifying full‐length or near full‐length proteins. Most notably a 28 kilodalton protein was isolated from rat liver (Nicholson et al., 1981) with sequence information for about 50 residues in the amino terminus. Later biochemical analyses revealed a nonidentical but related protein as the major constituent of rat heart gap junctions (Nicholson et al., 1985). These results supported immunological analyses in defining gap junction proteins of different vertebrate tissues as homologous (Bok et al., 1982; Dermietzel et al., 1984; Hertzberg and Skibbens, 1984). Connexin proteins were named according to their predicted molecular weight in kilodaltons (e.g., connexin26) with genes grouped according to sequence similarity. In early studies, vertebrate connexin genes were simply divided into alpha (α) and beta (β) groups based on sequence similarity (Kumar and Gilula, 1992). Additional subgroups have been added creating five subgoups (A through E) with connexin gene names beginning with “Gj” and connexin protein names beginning with “Cx” (Beyer and Berthoud, 2009). For instance Cx26 is encoded by the *Gjb2* gene, representing categorization as a gap junction protein of beta‐type and noting that it was the second beta connexin to be categorized.

### Innexins

Identification of the molecular components of invertebrate gap junctions unfolded primarily from forward genetic screens. Mutant flies (*Drosophila melanogaster*) and worms (*Caenorhabditis elegans*) were identified with phenotypes resulting from abnormal intercellular communication (Phelan, 2005). In early studies of *Drosophila*, behavioral, physiological, and developmental changes were noted after disruption of genes now known to encode for gap junction proteins (Wyman and Thomas, 1983; Ryerse and Nagel, 1984; Thomas and Wyman, 1984; Lipshitz and Kankel, 1985). For instance, Sun and Wyman (1984) noted that coupling between neurons in the Giant Fiber System (GFS) of *Drosophila* was disrupted in the mutant *Passover. Passover* was later found to be a transcript variant of the ShakingB locus and renamed ShakingB(neural) (*Zhang* et al., 1999).

By the 1990s *Drosophila* genes Ogre (optical ganglion reduced) and Shaking‐B were definitively correlated with gap junctions (Lipshitz and Kankel, 1985; Watanabe and Kankel, 1990, 1992; Crompton et al., 1995; Krishnan et al., 1995; Phelan et al., 1996; Sun and Wyman, 1996; Shimohigashi and Meinertzhagen, 1998; Blagburn et al., 1999). In *c. elegans*, the eat‐5 mutants which displayed asynchronous contraction of pharyngeal muscle cells and a loss of electrical and dye coupling, were identified as gap junction defects (Starich et al., 1996). Similarly, Unc‐7 mutants displaying defects in locomotion were correlated with gap junctions (Starich et al., 1993; Starich et al., 1996; Barnes and Hekimi, 1997).

The sequences of Ogre, Passover, Unc7, Unc9, and Shaking‐B were found to be very similar, leading to the distinction of a gene family known as OPUS because it included the genes ogre‐passover‐unc and shaking B (Barnes, 1994). The proteins encoded by OPUS genes had a membrane topology similar to that of connexins (Fig. 1, Membrane Topology) and it was speculated that they formed gap junctions (Barnes, 1994; Crompton et al., 1995; Starich et al., 1996). However, it was not until Phelan et al. (1998b) expressed ShakingB in *Xenopus* oocytes that it was conclusively deemed a gap junction protein distinct from connexins. Gap junction proteins of *c. elegans* were expressed in oocytes and also formed gap junctions (Landesman et al., 1999) and it became apparent that gap junctios were composed of different proteins in vertebrates and invertebrates (Phelan et al., 1998a).

The name innexin replaced OPUS (Phelan et al., 1998a) as the growing family of gap junction genes in *Drosophila* and *c. elegans* became apparent (Phelan and Starich, 2001). Innexins have since been identified in all invertebrate phyla with the exception of sponges and echinoderms (Phelan, 2005; Yen and Saier, 2007; Hasegawa and Turnbull, 2014). Innexin genes are also encoded in the genome of a parasitic wasp (Turnbull et al., 2005). Innexin homologs are found in the genome of vertebrates (Panchin et al., 2000) where they code for transmembrane rather than junctional channels (Sosinsky et al., 2011). Genes encoding connexins have not been found in invertebrates and there is no sequence homology between connexin and innexin genes (Alexopoulos et al., 2004; Phelan, 2005).

A major difference between innexin and connexin genes involves the positioning of introns (Fig. 1, Gene Structure). Introns are included in the coding region of innexin genes but not connexins (Phelan and Starich, 2001; Phelan, 2005). Hence, invertebrates are able to produce multiple gap junction proteins (splice variants) from the same gene while vertebrates are not. Innexins also have generally longer extracellular loops, and include two conserved cysteines in each loop (Phelan et al., 1998; Phelan and Starich, 2001; Phelan, 2005). In contrast, connexins have three conserved cysteines per loop (Beyer and Berthoud, 2009). The extracellular loops of innexins include glycosylation sites whereas connexins do not (Dahl and Muller, 2014; Calkins et al., 2015). Another interesting sequence comparisons is related to the positioning of a conserved proline in the second transmembrane domain of connexins that also appears to be present in all innexins (Phelan, 2005). In connexins, the proline may play a role in transduction of voltage‐gating (Cx26) (Suchyna et al., 1993) and in innexins has been associated with a cold‐sensitive phenotype (*ce* Unc‐9) (Barnes and Hekimi, 1997).

## FUNCTIONAL ASPECTS OF VERTEBRATE AND INVERTEBRATE GAP JUNCTIONS ARE REMARKABLY SIMILAR

**Figure 2:**
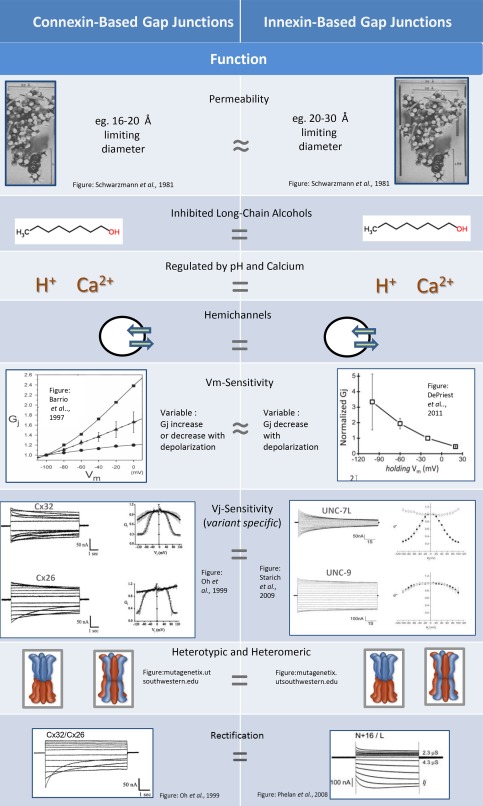
Comparison of gap junctions composed of connexins and innexins focusing on function. Permeability: Space‐filling models of a glycopeptide used to establish permeation‐limiting dimensions of gap junctions. Gap junctions of invertebrates were permeable to the larger version of the molecule (Left) while only the smaller version permeated mammalian junctions (Right) (Schwarzmann et al., 1981). Inhibited Long‐Chain Alcohols: Representative structure of 1‐octanol, a compound that inhibis gap junctions composed of connexins (Left) and innexins (Right). Long‐chain alcohols (Scemes et al., 2009), carbenoxlone (Bao et al., 2007) and arachidonic acid (Weingart and Bukauskas, 1998) also inhibit gap junctions from vertebrate and invertebrate tissue. Regulated by pH and Calcium: Gap junctions of vertebrates (Left) and invertebrates (Right) are known to be regulated by pH and calcium. Cytoplasmic acidification induces channel closure via conformational changes in cytoplasmic domains (Morley et al., 1996; Wang and Peracchia, 1998). Innexin‐based channels are also sensitive to pH (Giaume et al., 1980) but the mechanism is not understood. Coupling in vertebrate and invertebrate preparations is reduced by calcium ions (Lowenstein et al., 1967; Délèze and Loewenstein, 1976). Hemichannels: Some connexins (Left) and innexins (Right) function physiologically as half‐channels (hemichannels) mediating transport across the plasma membrane, a feature that does not seem to limit their ability to function as intercellular channels (reviewed by Ebihara, 2003; Bao et al., 2007). Vm‐Sensitivity: Intact gap junction channels may exhibit sensitivity to Vm as demonstrated for connexins (Left; Barrio et al., 1997)) and innexins (Right; DePriest et al., 2011). Vj‐Sensitivity: Under voltage clamp, junctional currents demonstrate unique properties in terms of time‐ and voltage‐dependence. Currents were recorded from oocytes expressing Cx32 [left top] and Cx26 [left bottom] (Oh et al., 1999) or Unc‐7L [right top] and Unc‐9 [right bottom] (Starich et al., 2009). Heterotypic and Heteromeric: Cartoon representing gap junction channels composed of different isoforms of connexins (Left) and Innexins (Right). Most native channels are likely to involve dynamic and complex interactions between protein isoforms (Koval et al., 2014). Rectification: Heterotypic combinations of Cx26/Cx32 (Left) and ShakB N + 16/ShakB L (Right) produce channels with properties of electrical rectification (Oh et al., 1999; Phelan et al., 2008).

The functional aspects of vertebrate and invertebrate gap junctions are remarkably similar. Comparable features are highlighted in Figure 2. Both vertebrates and invertebrates express multiple versions of species‐specific gap junction proteins in overlapping patterns. Expression patterns differ during stages of development and also change in response to environmental cues. Some gap junction proteins perform very specific roles where as others are expressed in a wide variety of tissues and cells (Reviewed by Willecke et al., 2002; Phelan and Starich, 2001).

Historically, many functional characteristics of gap junctions were apparent before morphological features were studied by electron microscopy. Early studies revealed electrical coupling between neurons (Furshpan and Potter, 1957; Bennett et al., 1963) and the association between electrical coupling and the morphological presence of gap junctions became apparent in a wide range of preparations including teleost neurons (Bennett et al., 1963; Barr et al., 1965), club endings of the Mauthner cell synapse of goldfish (Robertson, 1963) and crayfish neurons (Payton et al., 1969). The permeability of gap junctions to fluorescent molecules and metabolites was also revealed very early in the history of gap junction research (e.g., Loewenstein and Kanno, 1964). By the 1970s numerous studies had characterized the physiological features of gap junctions including electrical (ionic) coupling, dye permeability and metabolic coupling (Payton et al., 1963; Loewenstein and Kanno, 1964; Stoker, 1967; Subak‐Sharpe et al., 1969 Johnson and Sheridan, 1971; Sheridan, 1971). Within a few years a great deal of information was available regarding gap junction physiology, including information about pore features related to size and selectivity (e.g., Simpson et al., 1977; Flagg‐Newton et al., 1979; Brink and Dewey, 1980; Schwarzmann et al., 1981). Electrical and dye coupling unfolded as rapid and reliable independent methods for demonstrating the presence of gap junctions (Harris, 2001) contributing to the observation that gap junctions are present in virtually all tissues from all animals (Beyer and Berthoud, 2009).

Some of the most notable early functional assays included the following observations

- There is a very low resistance between cells coupled by gap junctions (Bennett et al., 1963; Loewenstein and Kanno, 1964; Payton et al., 1969). For instance preparations of epithelial tissue of the *Drosophila* salivary gland demonstrated low resistance between cells, with high resistance along the intercellular path (Loewenstein and Kanno, 1964). Loewenstein and Kanno reported that resistance along the chain of salivary gland cells was only slightly higher than that of cytoplasm, and that small ions move relatively freely between cells.
- Permeant molecules include metabolites and dyes (Subak‐Sharpe et al., 1969). In a study involving metabolically deficient mammalian cells in culture Subak‐Sharpe et al. (1969) showed that coculturing with metabolically healthy cells rescued their metabolically deficient counterparts, most likely due to transfer of small nucleotides or genetic information. In addition, the early study by Loewenstein and Kanno (1964) demonstrated relatively free diffusion of the dye sodium fluorescein (mol. wt. 376) between cells in the *Drosophila* salivary gland.
- There are differences in the size exclusion limits of vertebrate and invertebrate gap junctions. For instance Schwarzmann et al. (1981) conducted a detailed analysis of pore diameter using uncharged permeant sugar molecules tagged with a fluorescent molecule. Oligosaccharides and glycopeptides were synthesized to include a fluorescent tag (FITC) and the probes were injected into cells of insect and mammalian tissue. Probes were also tested on insect and mammalian cultured cells. Transfer of probes to adjacent cells suggested a channel diameter of 20–30 A° for invertebrate junctions (*Chironomus* salivary gland and cultured AC‐20 insect cells) compared to a cutoff in the range of 16–20 A° for mammalian gap junctions (cultured B, RL and 3T3‐BALB/c cells).
- The structural differences observed between vertebrate and invertebrate junctions had functional consequences in terms of communication specificity. Epstein and Gilula (1977) demonstrated that coupling occurred between different insect cells, or vertebrate cells, whereas virtually no coupling was observed between cells of phylogenetically distant species.when cell lines originating from diverse animal species were co‐cultured

These and other studies are described below with references and reviews listed in Table 2.

### Permeability

Gap junctions are generally described as dense arrays of channels allowing molecules up to about 1 kilodalton to pass freely between cells. In reality there is great variation in the size and types of molecules that permeate junctions from different animals and tissues. This is one reason investigators use molecular probes as a tool to better understand the unique properties of gap junction channels and/or properties imparted by heteromeric or heterotypic channels (reviewed by Phelan and Starich, 2001; Harris and Locke, 2007). Gap junctions of all sorts are permeable to ions and small metabolites, and most are permeable to a wide range of molecular probes. Assessing dye permeability is one of the most common methods of identifying cells coupled by gap junctions *in vivo* or in cell culture (e.g., Hanani, 2012; Decrock et al., 2016), often leading to detailed characterization of exogenously expressed channels (Weber et al., 2004) or a better understanding of channel regulation (Spray et al., 1979). Although there is great diversity in the cut‐off limit for molecular probes within vertebrates and invertebrates, gap junctions of invertebrates are generally permeable to larger molecules than those of vertebrates (Fig. 2, Permeability). This was demonstrated by Schwarzmann and colleagues (1981) using branched glycopeptides to establish permeation‐limiting dimensions of gap junctions from various species. Galactose attachments were systematically removed to create different sized molecules with similar properties. It was established that the gap junctions of invertebrates were permeable to larger versions of the molecule than mammalian junctions.

### Inhibited by Long Chain Alcohols

A wide range of pharmaceutical agents are known to modulate gap junction intercellular communication (Bodendiek and Raman, 2010). The earliest of these to be identified and characterized were long‐chain alcohols, which were found to inhibit gap junction function in diverse tissue and cell types (Johnston et al., 1980; Spray et al., 1985). Blockers were, and still are, commonly used to correlate physiological or morphological features with gap junction intercellular communication with consideration for the fact that most gap junction blockers are nonspecific, targeting multiple gap junction forms, and molecules unrelated to gap junctions (Juszczak and Swiergiel, 2009). A great number of gap junction blockers have been identified for vertebrate gap junctions, many of which have been tested on innexin‐based junctions and shown to exert similar effects. These include carbenoxolone, glycyrrhetinic acid, quinine, quinidine, mefloquine, heptanol, octanol, anandamide, fenamates, 2‐APB, several anaesthetics, retinoic acid, oleamide, spermine, aminosulfonates, and sodium propionate (Juszczak and Swiergiel, 2009). Molecules known to also inhibit innexin‐based channels include long‐chain alcohols (Fig. 2, Inhibited Long‐Chain Alcohols) such as heptanol and octanol (Johnston et al., 1980; Spray et al., 1985; Scemes et al., 2009), carbenoxlone (Bao et al., 2007), and arachidonic acid (Weingart and Bukauskas, 1998). Although the mechanisms by which these diverse compounds interact with gap junction channels are complex and often poorly understood it is interesting that the same molecules modify gap junction channels composed of different protein families. One study that may shed light on a common mechanism of action involves gap junction‐coupled insect cells. Weingart and Bukauskas (1998) showed that intercellular conductance was reduced by the application of lipophilic agents such as long‐chain *n*‐alkanols (*n*‐hexanol, *n*‐heptanol, *n*‐octanol, *n*‐nonanol, *n*‐decanol) or arachidonic acid and using biophysical analyses noted that the mechanism of inhibition was related to modification of a Vm (membrane‐potential)‐sensitive gate within the gap junction channel.

### Regulated by pH

Gap junction channels of both vertebrates and invertebrates are known to be regulated by pH (Fig. 2, Regulated by pH and Calcium). In connexin‐based channels cytoplasmic acidification induces channel closure via conformational changes in intracellular domains, namely the C‐terminus and/or cytoplasmic loop. While the exact mechanism appears to vary between members of the connexin family (Liu et al., 1993; Morley et al., 1996; Wang and Peracchia, 1998; reviewed by Harris, 2001), Cx43 was the first connexin found to gate via a “particle/receptor” mechanism (Morley et al., 1996). Low pH triggers interactions between the C‐terminus and part of the cytoplasmic loop. While it is well known that innexin‐based channels are sensitive to cytoplasmic pH (Giaume et al., 1980; Obaid et al., 1983; Moreno et al., 1991; Landesman et al., 1999; Anderson and Woodruff, 2001) the mechanism has not yet been studied.

### Regulated by Calcium

The influence of calcium ions on gap junction coupling has been evident for over five decades (reviewed by Spray et al., 1985; Harris, 2001) and was the basis of an early “calcium hypothesis” stating that cytoplasmic calcium ion levels regulate gap junction function (Loewenstein, 1966). The calcium ion is a ubiquitous molecule that plays important roles in regulating cell processes. It makes sense that increases in cytosolic calcium, which may be correlated with cell damage as well as necrotic and apoptotic cell death, would be used a mechanism of uncoupling cells (Loewenstein and Rose, 1978). Early on it was apparent that electrical coupling and dye coupling between diverse cell types, including those from vertebrate and invertebrate preparations, was reduced by calcium ions (Lowenstein et al., 1967; Délèze and Loewenstein, 1976; Rose et al., 1977; Baux et al., 1978; Flagg‐Newton and Loewenstein, 1979; Obaid et al., 1983) (Fig. 2, Regulated by pH and calcium). Despite significant efforts to identify the mechanistic changes underlying calcium‐induced changes in coupling, questions remain. Early structural studies revealed that gap junction channels displayed different conformations when gap junction plaques were prepared in the presence and absence of calcium ions (Unwin and Zampighi, 1980; Unwin and Ennis, 1984). The conformational changes observed by Unwin's group involved tilting and splaying of subunits surrounding the pore. Other studies revealed changes in channel height consistent with rearrangement of cytoplasmic domains (e.g., Müller et al., 2002) and very minor conformational changes in the pore‐lining (Bennett et al., 2016). All structural analyses related to calcium sensitivity have so far been performed on connexin‐based channels and it not known whether innexin‐based channels display similar complexity.

### Occasionally Function as Hemichannels

Some connexins and innexins function physiologically as half‐channels (hemichannels) mediating transport across the plasma membrane of cells (Fig. 2, Hemichannels). This does not seem to limit their ability to function as intercellular channels (reviewed by Ebihara, 2003; Bao et al., 2007). For instance Cx46 which is expressed in the vertebrate lens was one of the first connexins to be characterized as a transmembrane channel after expression in *Xenopus* oocytes (Paul et al., 1991; Ebihara and Steiner, 1993). The *Xenopus* oocyte expression system was also used to characterize the first innexin‐based hemichannels. Two leech innexins were confirmed to mediate transmembrane currents and speculated to mediate ATP release after injury to the CNS, analogous to the role of pannexin channels in vertebrates (Bao et al., 2007). The leech hemichannels were regulated by cytoplasmic acidification and were sensitive to carbenoxelone, two features associated with connexin‐channels (Bao et al., 2007).

**Table 2:** Representative Studies and Reviews Related to Functional Attributes Shared by Vertebrate and Invertebrate Gap Junctions

Functional Attribute	Vertebrate (Connexins) *Key papers and Reviews*	Invertebrate (Innexins) *Key papers and Reviews*
Multiple subunit types expressed in one organism	Traub and Willecke, 1982 Paul, 1995 Reviewed by Willecke et al., 2002	Curtin et al., 1999 Zhang et al., 1999 Reviewed by Phelan and Starich, 2001; Phelan, 2005
Expression patters are specific yet overlapping and regulated (e.g., during development). Some proteins are widely expressed while others are specialized.	Paul, 1995 **Reviewed** by Willecke et al., 2002	Todman et al., 1999 Curtin et al., 1999 Zhang et al., 1999 Starich et al., 2009 **Reviewed by** Starich et al., 2001 Phelan and Starich, 2001 Phelan, 2005
Facilitates ionic coupling	Bennett et al., 1963 Weidmann, 1966 Payton et al., 1969 Gilula et al., 1972 **Reviewed by** Harris and Locke, 2007	Furshpan and Potter, 1957 Loewenstein and Kanno, 1964 Ducret et al., 2006 Weng et al., 2008 **Reviewed by** Phelan and Starich, 2001 Phelan, 2005
Facilitates metabolic coupling and intercellular signaling	Subak‐Sharpe et al., 1969 Gilula et al., 1972 Bevans et al., 1998 Goldberg et al., 1999 **Reviewed by** Harris and Locke, 2007	Anderson and Woodruff., 2001 Ayukawa et al., 2012 **Reviewed by** Phelan and Starich, 2001 Phelan, 2005
Mediates transfer of dyes and molecular probes	Flagg‐Newton and Loewenstein, 1979 Schwarzmann et al., 1981 Elfgang et al., 1995 Valiunas et al., 2002 **Reviewed by** Harris and Locke, 2007 Hanani, 2012	Loewenstein and Kanno, 1964 Schwarzmann et al., 1981 Anderson and Woodruff, 2001 Ducret et al., 2006
Inhibited by long‐chain alcohols and similar molecules	Johnston et al., 1980 Spray et al., 1985 **Reviewed by** Bodendiek and Raman, 2010	Weingart and Bukauskas, 1998 Bao et al., 2007 **Reviewed by** Scemes et al., 2009
Sometimes plays a role in nonapposed membranes (e.g., hemichannels)	Paul et al., 1991 DeVries and Schwartz, 1992 Ebihara and Steiner., 1993 Cotrina et al., 1998 De Vuyst et al., 2006 **Reviewed by** Ebihara, 2003	Bao et al., 2007
Subunit interactions lead to formation of heterotypic channels	White et al., 1994 **Reviewed by** White and Bruzzone, 1996 Yeager, 2009 Koval et al., 2014	Stebbings et al., 2000 **Reviewed by** Phelan and Starich, 2001 Starich et al., 2001
Subunit interactions lead to formation of heteromeric channels	Jiang and Goodenough, 1996 Brink et al., 1997 Smith et al., 2012 **Reviewed by** White and Bruzzone, 1996 Koval et al., 2014	Phelan et al., 2008 Starich et al., 2009 **Reviewed by** Phelan and Starich, 2001 Phelan, 2005
Calcium regulates channel	Flagg‐Newton and Loewenstein, 1979 Unwin and Zamphigi, 1980 Unwin and Ennis, 1984 Müller et al., 2002 Bennett et al., 2016 **Reviewed by** Spray et al., 1985 Harris, 2001	Baux et al., 1978 Obaid et al., 1983 Bennett et al., 2016
pH regulates channel	Flagg‐Newton and Loewenstein, 1979 Campos de Carvalho et al., 1984 Ek‐Vitorín et al., 1996 **Reviewed by** Harris, 2001 Spray et al., 1985 Lewandowski et al., 2007	Giaume et al., 1980 Obaid et al., 1983 Moreno et al., 1991 Landesman et al., 1999 Anderson and Woodruff, 2001
Transjunctional voltage (Vj) regulates channel	Spray et al., 1979 Harris et al., 1981 **Reviewed by** Spray et al., 1985 Harris, 2001 Bargiello and Brink, 2007	Obaid et al., 1983 Verselis et al., 1991 Chanson et al., 1994 Phelan et al., 1998 Landesman et al., 1999 Starich et al., 2009 DePriest et al., 2011 Marks and Skerrett, 2014
Transmembrane voltage (Vm/V_i‐o_) regulates channel	Spray et al., 1979 **Reviewed by** Spray et al., 1985 Harris, 2001 Bargiello and Brink, 2007	Obaid et al., 1983 Verselis et al., 1991 DePriest et al., 2011

#### Vm Sensitivity

Voltage regulation is generally divided into two categories—regulation by transmembrane voltage (Vm or Vi‐o) and regulation by transjuntional voltage (Vj). Vm‐sensitivity indicates that junctional conductance is dependent on the membrane potential of the coupled cells. This phenomenon appears to be common for connexin‐ and innexin‐based junctions (Verselis et al., 1991; reviewed by Phelan and Starich, 2001; see Table 2) but was first characterized in invertebrate preparations (Verselis et al.,^1991^; Bukauskas et al., 1992). The gap junctions between insect cells exhibited high sensitivity to Vm with conductance decreases in response to depolarization (Fig. 2, Vm‐Sensitivity). Weaker Vm‐sensitivity was later noted for vertebrate junctions where there appears to be great variation in the response to holding potential, with responses ranging from conductance that decreases with depolarization (Barrio et al., 1993; White et al., 1994) to conductance that increases with depolarization (Barrio et al., 1991). One of the most interesting findings regarding Vm sensitivity comes from a unique study involving four connexin homologs (Cx45 from zebrafish, chicken, mouse, and human) expressed in *Xenopus* oocytes. It was shown that the Vm‐sensitive channel gate functions independently of the Vj‐gate and that each hemichannel in a gap junction contains an independent Vm‐gate. As well as characterizing the relationship between Vj‐ and Vm‐dependent gating, the study provided evidence that voltage gating properties diverged during vertebrate evolution (Barrio et al., 1997). Biophysical analysis of insect junctions confirms that the Vj and Vm gates are also independent in innexin‐based channels (Verselis et al., 1991)

### Vj‐Sensitivity

Vj‐sensitivity is an interesting biophysical phenomenon because it requires the channel to sense and respond to a voltage differences across the junction, a feature unique to gap junction channels (Spray et al., 1981). All connexins and innexins identified so far exhibit sensitivity to Vj although in many cases the response is minor and/or not likely to have physiological significance. Each gap junction protein (connexin or innexin) appears to impart a unique time‐ and voltage‐dependent response to Vj (Fig. 2, Vj‐Sensitivity, Variant Specific). For instance Cx26 and ShakingB(neural + 16) are relatively insensitive to Vj, whereas Cx43 and *ce*‐Unc9 are quite voltage‐sensitive (Harris, 2001; Phelan et al., 2008; Starich et al., 2009). Characterization of Vj‐sensitivity requires that opposing sides of the junction are voltage‐clamped so that transjunctional voltage can be controlled (Harris et al., 1981; reviewed by Harris, 2001).

Vj‐sensitivity is one of the most well characterized aspects of gap junction function and there is a large body of literature and review literature on the topic (Spray et al., 1979; reviewed by Spray et al., 1985; Harris, 2001; Bargiello and Brink, 2007). Some aspects that have been studied include; conductance versus Vj relationships (Spray et al., 1979; Veenstra, 1990; Rubin et al., 1992; Valiunas et al., 1997), independent nature of the Vj‐gate (Barrio et al., 1997; Harris et al., 1981; Verselis et al., 1991), gating polarity (Rubin et al., 1992; Oh et al., 1999), implications of Vj‐gating on electrical rectification (Jaslove and Brink, 1986; Oh et al., 1999; Phelan et al., 2008), interactions between the Vj‐gate and other gates (Barrio et al., 1997; Valiunas et al., 1999), conductance states and permeability of Vj‐gated channels (Spray et al., 1979; Valiunas et al., 1997), structural determinants of the Vj gate (Rubin et al., 1992; Suchyna et al., 1993), structural determinants of the Vj sensor (Oh et al., 2000). Most of these studies involved connexin‐based channels; however, in a few cases where Vj‐gating of innexin‐based channels has been studied there are strong similarities to connexin‐based channels (Obaid et al., 1983; Jaslove and Brink, 1986; Verselis et al., 1991; Chanson et al., 1994; Phelan et al., 1998; Landesman et al., 1999; Starich et al., 2009; DePriest et al., 2011; Marks and Skerrett, 2014).

### Heterotypic and Heteromeric Channels

Most animal cells express multiple gap junction proteins (either connexins or innexins) allowing many potential interactions. Interactions between gap junction proteins commonly result in heteromeric and/or heterotypic gap junction channels (Fig. 2, Heterotypic and Heteromeric) both of which are common in vertebrate and invertebrate systems (White et al., 1994; Jiang and Goodenough, 1996; Brink et al., 1997; Stebbings et al., 2000; Phelan et al., 2008; Starich et al., 2009; Smith et al., 2012)**.** Heteromeric interactions involve the oligomerization of two or more isoforms within a half‐channel whereas heterotypic interactions involve interactions between different proteins in adjacent cells (reviewed by Koval et al., 2014). The potential for such interactions is often assessed after exogenous expression (Skerrett et al., 2000; Phelan et al., 2008; Starich et al., 2009; Koval et al., 2014) where characteristics such as gating, permeability and regulation reveal properties that differ from those of either of the single contributors. It is also possible to examine interactions biochemically, genetically, or morphologically (Koval et al., 2014). However, methods for assessing interactions are time‐consuming and require knowledge of the expected interaction. As such, oligomerization and interactions remain two of the most poorly characterized physiological aspects of gap junction function. This presents a challenge in understanding gap junction‐mediated intercellular communication because most native channels are likely to involve dynamic and complex interactions between protein isoforms.

### Rectification

Heterotypic interactions occasionally lead to junctional rectification (reviewed by Palacios‐Prado et al., 2014), a rare physiological phenomenon observed with connexin‐based junctions (Oh et al., 1999) and innexin‐based junctions (Phelan et al., 2008) (Fig. 2, Rectification). Furshpan and Potter (1957) were the first to note asymmetry in the transmission of electrical signals between coupled neurons within the Giant Fiber System (GFS) of crayfish. Further characterization confirmed that the synapse acted as an electrical rectifier favoring transmission of depolarization toward the postsynaptic cell (Furshpan and Potter, 1959) a characteristic attributed to asymmetric voltage‐sensitivity of gap junction channels at the synapse (Jaslove and Brink, 1986). However, asymmetry in this system is “instantaneous,” occurring too rapidly to rely on typical Vj‐dependent gating. Further characterization at the single channel level is required to establish whether rapid electrical rectification occurs as a result of asymmetry in conduction or fast Vj‐gating events unresolved at the level of macroscopic recordings. Rapidly rectifying electrical synapses have also been identified in the GFS of *Drosophila* (Margiotta and Walcott, 1983; Phelan et al., 1996; Allen et al., 2006) and are now known to result from heterotypic interactions between different variants of the ShakingB locus (Phelan et al., 2008). The ability to recreate rectifying synapses after exogenous expression (Phelan et al., 2008) and modify innexins in structure‐function analyses (Marks and Skerrett, 2014) should facilitate single channel analysis leading to a more thorough understanding of rectification.

Electrical rectification also occurs in chordates (reviewed by Palacios‐Prado et al., 2014) including neural circuits involved in escape responses (Auerbach and Bennett, 1969; Ringham, 1975; Rash et al., 2013). The most well‐characterized vertebrate junction of this type is found at Mauthner cell club endings of the goldfish where is has been shown that homologs of the vertebrate neuronal connexin Cx36 (namely fish Cx34.7 and fish Cx35) form heterotypic junctions (Rash et al., 2013). In this system, characterization has focused on understanding the complexities of mixed synapses (chemical/electrical) and the physiological consequences of rectification that favors antidromic transmission, acting as a mechanism of lateral excitation (Pereda et al., 1995; Rash et al., 2013; reviewed by Palacios‐Prado et al., 2014). The detailed characteristics of these heterotypic junctions under voltage‐clamp have not been reported.

A number of studies of rectification at the molecular level have focused on channels composed of Cx26/Cx32. These heterotypic junctions display rectification related to different Vj‐sensitivity, namely opposite polarity of the Vj‐sensor (Verselis et al., 1994, reviewed in Harris, 2001) as well as “instantaneous” rectification that appears to result from asymmetry of charges within or near the channel pore (Rubin et al., 1992; Oh et al., 1999; Suchyna et al., 1999). Characteristics of Cx32/Cx26 junctions under voltage clamp are included in Figure 2.

In summary, functional assays reveal remarkable similarity between vertebrate and invertebrate gap junctions. Both impart low resistance connections between cells with defined permeability and selectivity for large molecules. Common factors regulate gap junction channels, such as pH, calcium, and transjunctional voltage. A few connexins, and few innexins are known to form hemichannels that function in nonapposed membranes. In cases where vertebrate and invertebrate junctions are carefully compared, differences in the cut‐off limit for permeant molecules appears to be the prevailing functional distinction.

## THREE DIMENSIONAL MODELS DEMONSTRATE STRUCTURAL DIFFERENCES BETWEEN CONNEXIN‐ and INNEXIN‐BASED GAP JUNCTION CHANNELS

The first three‐dimensional structure of a gap junction channel was obtained from a mammalian liver preparation in 1980 (Unwin and Zampighi, 1980). Since that time, connexin channel structures have become progressively more refined (e.g., Cx43, Unger et al., 1999; Cx26, Oshima et al., 2007; Cx26 Maeda et al., 2009; Cx26 Bennett et al., 2016). All models of connexin channels reveal six subunits evenly spaced around a central pore, a dodecameric channel consisting of two six‐subunit rings with extracellular domains locked together (Yeager and Harris, 2007). The earliest structural studies produced low resolution maps using electron microscopy enhanced by negative stain (Unwin and Zampighi, 1980), X‐ray scattering (Unwin and Ennis, 1983), and cryo‐electron microscopy (Unwin and Ennis, 1984). These revealed a central pore of about 20 Å in diameter, surrounded by six subunits, with each subunit occupying an area of about 25 Å diameter. The extracellular regions of the gap junction channel are correlated with an intercellular gap of about 40 Å. The four membrane‐spanning domains were predicted to have alpha‐helical secondary structure (e.g., Milks et al., 1988) but this was not confirmed structurally until a higher resolution projection structure was obtained (Unger et al., 1997).

Subsequent three dimensional projection structures involving reconstituted proteins reveal remarkably similar images of Cx43 (*truncated at C‐terminus amino acid 263*; Unger et al., 1999) and Cx26 (*M34A mutant*; Oshima et al., 2007). The unit diameter of the Cx43 channel is about 150 Å, 100 Å less than observed in EM images of native channels presumably due to truncation of the C‐terminus domain (Unger et al., 1999). The pore of the channel (side chains excluded) is about 40 Å wide at the cytoplasmic mouth, narrowing to 15 Å at the extracellular mouth, and widening again within the extracellular space to about 25 Å. The Cx26 structure reveals almost identical channel dimensions and pore diameter when superimposed on the Cx43 structure. Positions of the transmembrane helices were also very similar and minor variations in the transmembrane domain positions were attributed to different crystallization procedures rather than real differences between channels composed of different connexins (Oshima et al., 2007). The Cx26 structure revealed a plug in the vestibule, most likely formed by the amino terminus folded into the mouth of the pore (Oshima et al., 2007; Oshima et al., 2008). An in‐folded amino terminus was also present in the atomic model of a Cx26 channel obtained using X‐ray crystallography with resolution of up to 3.5 Å (Maeda et al., 2009). At this higher resolution, the N‐terminus is observed closely interacting with the pore‐lining helix (TM1) at the mouth of the pore but does not form a prominent density in the middle of the pore. These differences may be related to an alternate conformational state of the channel (e.g., open versus closed) related to the M34A mutation (Oshima et al., 2007).

Cx26 has also been studied using X‐ray crystallography by Bennett et al. (2016) in calcium‐bound and unbound states. The general channel structure is almost identical to the structure of Maeda et al. (2009). This structure addresses the mode of calcium‐dependent regulation of gap junctions and with the assistance of computer modelling demonstrates that calcium inhibits channel conductance by binding within the pore and inducing minor conformational changes associated with an electrostatic barrier to ions. These changes may occur in addition to, or in contrast to larger conformational rearrangements observed in lower resolution X‐ray and EM structures (Unwin and Zampighi, 1980; Unwin and Ennis, 1983; Unwin and Ennis, 1984) and AFM analysis of gap junctions (Müller et al., 2002) where calcium‐induced conformational changes are consistent with tilting and splaying of transmembrane helices and/or an increase in channel length.

Following the first atomic model of connexin channels (Maeda et al., 2009) molecular dynamics simulations (e.g., Kwon et al., 2011; Kwon et al., 2012; Araya‐Secchi et al., 2014; Tong et al., 2014; Zonta et al., 2014; Luo et al., 2016) and other types of modeling including homology models (Brennan et al., 2015) and models of heterotypic channels (Gong et al., 2013) have been applied to connexin channels. These models have refined information about the gating states of the channel, pore dimensions, permeability and connexin interactions within a channel. To date, computer models have not been used to better understand the single channel behavior of innexin channels because this requires a three dimensional model with resolution suitable for side‐chain assignments.

**Figure 3:**
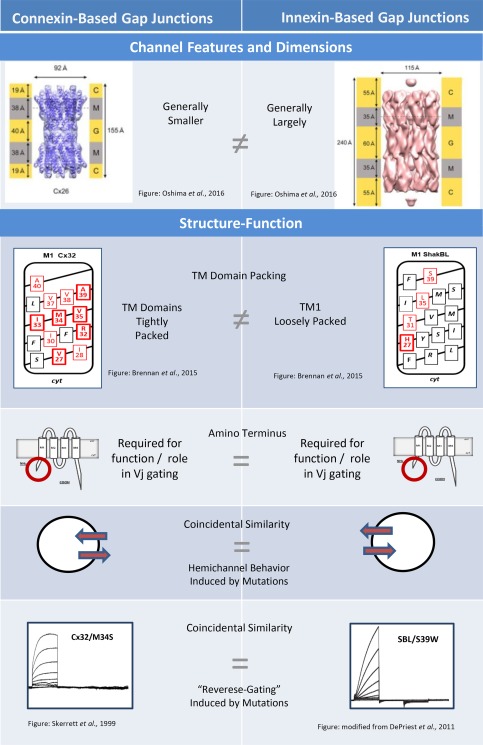
Comparison of gap junction channels composed of connexins and innexins focusing on channel features and structure‐function analysis. Channel Features and Dimensions: Surface view structures of gap junction channels composed of Cx26 (Left) and INX‐6ΔN (Right). Scales alongside the channels indicate length of transmembrane (M), intracellular© and extracellular (G/gap) regions. The Cx26 channel is approximately 155Å in length with an outside diameter of 92Å (Maeda et al., 2009). The INX‐6 channel is approximately 240Å in length with an outside diameter of 115Å (Oshima et al., 2016). Images from Oshima et al., 2016. TM Domain Packing: Helical net plots showing residues where tryptophan substitution rendered channels nonfunctional during tryptophan scanning in Cx32 (Left) and ShakBL (Right). Cx32 TM1 was highly sensitive to tryptophan substitution indicative of tight packing (Brennan et al., 2015) whereas only a few sites were sensitive to tryptophan substitution in TM1 of ShakBL (DePriest et al., 2011). These results are consistent with structural data indicating that innexin‐based channels are larger and involve more subunits than their connexin‐based counterparts (Oshima et al., 2016). Amino Terminus: Membrane topology highlighting importance of the amino terminus (NT). In both connexin‐(Left) and innexin‐based channels (Right) the amino terminus is required for function and plays an important role in Vj‐dependent gating. In connexins, the NT is 22–23 amino acids in length including a short α‐helix. The NT likely folds into the pore, lining part of the conduction pathway, consistent with its involvement in permeability, conductance and Vj‐gating (reviewed by Beyer et al., 2012). Innexins also appear to require an NT which also plays a role in Vj‐gating and rectification (Marks and Skerrett, 2014). Coincidental Similarities: Two similarities were noted in structure‐function studies, aberrant hemichannel behavior (arrows showing transport across cell membrane) and a “reverse‐gating” phenotype. Currents recorded from “reverse‐gating” channels M34S in Cx32 (Left; Skerrett et al., 1999) and S39W in ShakBL (Right; DePriest et al., 2011) are shown. A characteristic of “reverse‐gating” mutants is that they form channels that remain predominantly closed (or in a low conductance state) at Vj = 0 mV but open with higher Vj. Currents are often only apparent in heterotypic pairings with wildtype.

Only two studies have specifically targeted the structure of innexin‐based channels. These both relate to junctions composed of *c. elegans* INX‐6 using cryo‐EM (Oshima et al., 2013; Oshima et al., 2016). The first study provided channel dimensions using thin section and negative stain EM after expression of INX‐6 in Sf9 cells. The results confirmed early structural studies suggesting that innexin channels have a larger overall structure than connexin‐based channels (Oshima et al., 2013). Channel height, width and spacing were all considerably greater for INX‐6 channels than for Cx26 and Cx43‐GFP gap junction channels exogenously expressed in the same study (Fig. 3, Channel Features and Dimensions). For instance the junctional membrane width was about 184 Å for INX‐6 channels compared to 140 Å and 162 Å for Cx26 and Cx43‐GFP channels, respectively. The distance between channels (*en face*) assuming hexagonal packing was estimated to be 111 Å for INX‐6 channels compared to 94 Å and 77 Å for Cx26 and Cx43‐GFP, respectively. Oshima et al. (2013) noted that while INX‐6 channels appeared to be arranged in a hexagonal lattice, care should be taken in assigning an oligomeric number to innexin channels.

The structure of INX‐6 channels was later resolved at about 10 Å resolution using cryo‐electron microscopy. The amino terminus was truncated and the proteins were expressed, purified and crystalized. As predicted the pore was found to be wider than that of connexin‐based channels and the gap junction channel itself was both wider and longer (Oshima et al., 2016). The end‐to‐end length of the channel is roughly 240 Å (compared to 150 Å for Cx26 channels, Maeda et al., 2009), the pore diameter is about 40 Å (compared to 30 Å for Cx26 channels, Maeda et al., 2009), and the outer diameter of the channel is about 115 Å (compared to 92 Å for Cx26 channels, Maeda et al., 2009). The innexin channel is composed of 16 INX‐6 subunits, eight subunits surround the central pore of each hemichannel creating a hexadecameric channel. Perhaps the most interesting feature is the presence of two densities within the pore although it is difficult to assign any residues to the plug and bobble densities due to the limited resolution (Oshima et al., 2016).

An in‐folded CT and complex pore structure for INX‐6 channels may be supported by pore‐lining analysis of a close relative, the Panx1 channel (Wang and Dahl, 2010). It is likely that pannxins, which function as nonjunctional channels in chordates, share structural features with their evolutionary relatives the innexins. The conduction pathway of Panx1 was found to include residues at the extracellular end of M1 and the carboxyl terminus. The study tested accessibility of substituted cysteines and found several adjacent residues in TM1 and many residues in the CT were accessible and consistent with a pore‐lining location (Wang and Dahl, 2010). The pore‐lining of pannexin channels seems to be complex but is so far consistent with limited knowledge of INX‐6 pore structure (Oshima et al., 2016).

## STRUCTURE‐FUNCTION STUDIES ALLOW COMPARISON OF CONNEXIN‐ and INNEXIN‐BASED GAP JUNCTION CHANNELS

Structure‐function studies of innexins have so far focused on the *Drosophila* ShakingB innexins (Phelan et al., 2008; DePriest et al., 2011; Marks and Skerrett, 2014) expressed in *Xenopus* oocytes. Three transcript variants of the ShakingB gene are known; Shaking‐B(Lethal), Shaking‐B(Neural), and Shaking‐B(Neural + 16). Shaking‐B(Neural) does not readily form functional channels on its own (Phelan and Starich, 2001) and has not been studied at the structure‐function level. The other two proteins have been expressed exogenously and are commonly referred to as ShakBL (SBL) and ShakBN16 (SBN16). ShakBL was the first innexin to be exogenously expressed in oocytes (Phelan et al., 1998), was later shown to form rectifying junctions in oocytes when paired heterotypically with ShakBN16 (Phelan et al., 2008) and was the first subject of structure‐function analysis (DePriest et al., 2011).

As highlighted in Figure 3 (Structure‐Function) three important findings have so far come from structure‐function studies of innexins. The first structure‐function analysis provided straightforward information about TM domain packing in ShakBL (DePriest et al., 2011). The second structure‐function analysis focused on rectifying junctions formed by ShakBL/ShakBN16. Mutations were focused in the amino terminus (NT), interpretation was not straightforward, but again mutants displayed properties that arbitrarily correlated with observations for connexin‐based channels (Marks and Skerrett, 2014). Some of the mutants created for these studies coincidentally induced phenoptyes commonly observed in structure‐function studies of connexins.

### ShakBL and TM Domain Packing

In 2011, DePriest et al. studied TM1 of ShakBL using tryptophan scanning analysis. Tryptophan scanning mutagenesis is commonly applied to membrane proteins to gain a better understanding transmembrane domains interactions. It is based on a simple premise that the large bulky side‐chain of tryptophan will disrupt protein function if substituted at a site where helices closely interact (Sharp et al., 1995). Clearest results are obtained when one face of a transmembrane helix is revealed to be particularly sensitive to tryptophan (Ueno et al., 2000; Guzman et al., 2003; Ishii et al., 2007).

The tryptophan scan of TM1/ShakBL produced clear results in the sense that tryptophan substitutions disrupted gap junction function at only four sites, all of which lie on the same helical face (DePriest et al., 2011). Figure 3 (TM Domain Packing) highlights the amino acids sensitive to tryptophan substitution in ShakBL (H27, T31, L35, and S39). Although further work is required to determine if the interactions occur between or within subunits, the results demonstrate loose packing relative to connexins (Brennan et al., 2015).

In terms of comparing the structure of gap junctions formed by innexins and connexins, the tryptophan scanning data is particularly relevant when compared to results of tryptophan scanning in Cx32, where over 50% of residues in TM1 were sensitive to tryptophan substitution (Fig. 3 Right) (Brennan et al., 2015). Loose packing of TM helices is consistent with greater channel spacing in invertebrate preparations (Flower, 1971; Peracchia, 1973; Flower, 1977; Ohta et al., 2011) and with the generally larger channel reported for innexins (Oshima et al., 2013; Oshima et al., 2016).

### General Importance of the Amino Terminus

Structure‐function analysis of innexins has also focused on the amino terminus (NT) with the goal of identifying the role of this domain in voltage gating and junctional rectification (Marks and Skerrett, 2014). In the *Drosophila* giant fiber system, adjacent cells express and contribute different transcript variants of the innexin Shaking B, facilitating heterotypic ShakBN16/ShakBL gap junctions. The heterotypic synapse was recreated in *Xenopus* oocytes by Phelan et al. (2008), a study that in itself provided some interesting structure‐function information. Being splice variants of the Shaking‐B locus, ShakBN16 and ShakBL have identical amino acid sequences from the extracellular end of TM2 to the end of the CT. It is therefore apparent that properties unique to either of the innexins, including those leading to rectification, reside in the first one‐third of the protein. Several mutants were created in the study of Marks and Skerrett (2014) each yielding some information about the structural requirements for channel function and gating.

Several observations support the hypothesis that some form of an amino terminus (NT) is required for innexin function (Fig. 3 Amino Terminus). To assess the role of the NT in gating of ShakB innexins, the NT of ShakBL was removed creating a deletion mutant SBL*NTdel* (missing residues L2 through S21). This mutant failed to form functional homotypic channels (Marks and Skerrett, 2014) and when paired heterotypically with wtShakBL produced currents only slightly above background (Gj = 0.2 ± 0.1 μS). It has also been noted that Shaking‐B(Neural), which is identical to ShakBN16 but lacks the first 16 amino acids, fails to form functional channels (Phelan and Starich, 2001). INX‐6 channels from *c. elegans* involving an N‐terminal deletion (*18 deleted residues including amino acids 2 through 19*), while amenable to crystallization, also fail to function in dye‐transfer assays (Oshima et al., 2016). Taken together these three studies provide considerable evidence that, like their connexin counterparts, innexins require an intact NT to form functional channels. In connexin‐based channels NT deletions and other significant modifications to the NT render channels nonfunctional (Harris, 2001) and detailed analysis of NT requirements in Cx37 suggest that large or complete deletions disrupt trafficking and oligomerization while small modifications prevent properly formed channels from functioning (Kyle et al., 2008; Kyle et al., 2009).

### Role of the Amino Terminus in Gating and Rectification

To assess the role of the NT in voltage gating and rectification, the NT of ShakBL was replaced with that of ShakBN16 (the resulting mutant was termed SBLNTN16). The NT replacement resulted in rectifying junctions with properties similar to those of ShakBL/ShakBN16 junctions (Marks and Skerrett, 2014). In the absence of further inspection these results could be interpreted as evidence that properties of voltage gating and rectification are conferred by the first 22 amino acids (the NT).

However, further analysis provided evidence that voltage gating of innexins, at least the ShakB innexins, is complex. While the mutant SBLNTN16 behaved almost identically to ShakBN16 in heterotypic pairings, it behaved more like ShakBL when paired homotypically with itself. In addition, a complementary mutant, ShakBN16 with the NT of ShakBL (SBN16 NTL) did not induce the predicted response in heterotypic pairing with ShakBL The NT of ShakBL failed to confer properties of voltage‐gating and rectification to ShakBN16 channels. These observations ruled out the possibility that the NT of ShakB innexins mediates Vj gating independently (Marks and Skerrett, 2014). One explanation for these interesting results is that the innexins possess a gating mechanism determined both by the main body of the channel, and a voltage sensor carried in the NT. Other plausible explanations involve interactions between NT domains from apposing innexons and influence of other structural features, such as pore diameter, on voltage gating.

Overall the results of structure‐function analyses involving NT domain swaps in ShakB innexins provide evidence that Vj‐gating is complex and involves multiple domains. Although only two innexins were targeted for investigation the results suggest that gating mechanisms are somewhat innexin‐specific. Based on these limited analyses, it appears that in terms of Vj‐gating, innexins are similar to their connexin‐based counterparts. Numerous studies have provided details about the role of the connexin NT domains in voltage gating and rectification (reviewed in Harris, 2001) and a few studies have investigated complete replacement of NT domains. In one study the NT of chicken Cx45.6 was replaced with that of rat Cx43 (Dong et al., 2006). Resulting junctions displayed Vj‐gating similar to that of rat Cx43, and suggesting that in at least some cases Vj gating is independently carried by the NT. However, NT domain swaps in Cx32 and Cx26 suggest that the NT does not independently confer properties of Vj gating (Oh et al., 1999). Hence, it appears that the NT functions differently in different connexins or that interactions between the NT and another domains are required for *V*_j_ gating.

### Coincidental Observations Related to Point Mutations in TM1 of ShakBL

The tryptophan scanning analyses described above provide compelling evidence for structural differences between connexins and innexins, but interestingly, also identified interesting similarities in terms of mutant phenotypes (DePriest et al., 2011). Tryptophan substitutions in ShakBL were found to induce three phenotypes two of which are commonly observed in connexin mutants. Aberrant hemichannel behavior and a reversed response to transjunctional voltage (Vj) are common consequences of point mutations within the transmembrane domains of connexins *(*Fig. 3 *Coincidental Similarities)*. Altered sensitivity to transmembrane voltage (Vm‐sensitivity) was also apparent after one mutation in TM1 of ShakBL but is not discussed further because it is not a phenotype commonly noted in studies of connexins.

Aberrant hemichannel behavior has been observed for disease‐associated point mutations in many connexins (e.g., Cx26, Cx30, Cx31, Cx32, Cx40) (Retamal et al., 2015) and also results from point mutations associated with cysteine‐ and tryptophan scanning analyses (Skerrett et al., 2002; Brennan et al., 2015). Point mutations that induce currents in nonapposed membranes are distributed in several connexin domains including the amino terminus (NT), the extracellular end of TM1 (TM1/E1 border), the cytoplasmic loop (CL), and cytoplasmic tail (CT) (Retamal et al., 2015). Although structure‐function studies of innexins are currently restricted to the NT and TM1, similarity to connexins is apparent. One mutation (F24W) at the NT/TM1 border of ShakBL was also found to induce currents in nonapposed membranes (DePriest et al., 2011). Unusual membrane currents also resulted from point mutations at the extracellular end of TM1 with F38W and S39W mediating currents in nonapposed membranes (DePriest et al., 2011). Not only is the resulting change in function interesting due to its similarity to a functional consequence of point mutations in connexins, the involvement of residues at the TM1/E1 boundary is notable. In connexins, residues in this region *(≈ amino acids 42‐51)* form a short parahelix (Maeda et al., 2009), face the pore lumen (Maeda et al., 2009), play a role in voltage gating (e.g., Kwon et al., 2012), and regulate calcium binding (Bennett et al., 2016).

Although TM2 of innexins has not yet been the subject of structure‐function analysis, mutations inducing aberrant hemichannel behavior in connexins often occur in the mid‐region of TM2 and it will be interesting to determine if mutations in this region produce similar effects in innexin‐channels. For instance S85C is a well characterized CMTX mutation in Cx32 (Abrams et al., 2001) and A88V is a well characterized KID mutation in Cx30 (Mhaske et al., 2013). Other point mutations inducing leaky membranes include Cx32A88C (Skerrett et al., 2002) and Cx40V85I (Sun et al., 2014). These results suggest that important structural features related to channel regulation also occur in TM2. This is likely related to the presence of a conserved proline in the mid‐region of the TM2 helix, the local environment of side‐chains and their potential interactions with other TM domains (Maeda et al., 2009; Brennan et al., 2015). Residues in this region, particularly those corresponding to Cx26V84 and Cx26A88 face a putative water pocket (IC pocket) between the transmembrane helices that may play a role in gating (Araya‐Secchi et al., 2014). Given the presence of proline in TM2 of connexins and innexins, it will be interesting to determine if mutations in this region produce similar effects in innexin‐channels.

**A “reverse‐gating” phenoptype** also results from point mutations in connexins and innexins. As shown in Figure 3 for Cx32M34S and SBLS39W, mutations of this type induce a reversed response to transjunctional voltage (Suchyna et al., 1993; Oh et al., 1997; Skerrett et al., 1999; Abrams et al., 2001; Skerrett et al., 2002; Skerrett et al., 2004; Brennan et al., 2015). This phenotype is characteristically observed when a mutant is paired heterotypically with a wildtype gap junction protein and the term “reverse‐gating” emphsizes the tendency for currents to activate rather than inactivate in response to transjunctional voltage (Vj). This response was first observed by Suchyna et al (1993) who interpreted the response as an indication that the conserved proline in TM2 was essential for voltage gating. Further studies revealed that disease‐causing mutations associated with β‐type connexins such as M34T, V35M, and V38M in Cx32 (Oh et al., 1997) and M34T in Cx26 (Skerrett et al., 2004) also induce the phenotype. In scanning mutagenesis studies, point mutations at several sites in each TM domain of Cx32 induce reverse‐gating (Skerrett et al., 2002; Brennan et al., 2015). So far the “reverse‐gating” phenotype has only been observed with one innexin mutant, ShakBL S39W (DePriest et al., 2011). This mutant also displayed sensitivity to transmembrane voltage (DePriest et al., 2011).

Considerable effort has been placed on understanding one particular group of “reverse‐gating” mutants, those associated with mutations at position 34(methionine) in Cx32 and Cx26 (Oh et al., 1997; Skerrett et al., 1999; Skerrett et al., 2002; Skerrett et al., 2004). The “reverse‐gating” mutants represent a stabilized closed/low conductance state of gap junction channels (Skerrett et al., 2002) and the potential of such mutants for crystallization was harnessed using the Cx26M34A. This mutant was studied by cryo‐EM in 2007 (Oshima et al., 2007) providing the first evidence that a plug may exist in the vestibule of connexin‐based channels. Since there does not appear to be a plug in the vestibule of channels composed of wtCx26 (Maeda et al., 2009; Bennett et al., 2016) and amino terminal deletions alter its appearance (Oshima et al., 2008) it is easy to speculate that the NT acts as a gating plug, and with position modulated by the application of Vj. Given the diverse set of mutations capable of inducing the phenotype it seems likely that general destabilization of the channel could alter critical interactions between the NT and TM1 at the mouth of the pore, resulting in a splaying of the NT helices that are typically folded into the pore. Support for the general destabilization hypothesis is evident in structure‐function studies showing that “size matters” at position M34 in Cx32 with smaller side‐chains such alanine, cysteine, serine, and threonine resulting in a “reverse‐gating” phenotype, while leucine and glutamine substitutions maintain wild‐type gating (Skerrett et al., 1999). Molecular dynamics simulations of Cx26 suggest that the M34T substitution significantly reduces channel conductance by disrupting hydrophobic interactions between M34 and the NT (Zonta et al., 2014), consistent with single channel analysis demonstrating that Cx32M34T channels reside predominantly in a low conductance state (Oh et al., 1997). Continued research involving structural models, simulations, and structure‐function analyses will be required to uncover the molecular mechanisms of “reverse‐gating” and the information will be important for understanding a number of human diseases as well as for comparing structure and gating mechanisms of connexin‐ and innexin‐based channels. Currently it appears as though innexin‐based channels can be destabilized in a manner similar to that of connexin channels resulting in a “reverse‐gating” phenotype.

## SUMMARY

Given their nonhomologous genetic origins, innexins and connexins are remarkably similar. They have the same membrane topology. They oligomerize around a central pore to form a channel permeable to small molecules and ions. Innexins and connexins are the only known proteins to form intercellular channels, a feat that requires complex recognition and docking interactions to produce a tightly sealed channel spanning the extracellular space. While the molecular interactions underlying these process are poorly understood, it is interesting to note that connexins contain three conserved cysteines in each extracellular loop while innexins contain two. The intercellular channels produced by different members of the innexin and connexin families have unique properties, attesting to the need for specialized transport between cells of different tissues and organs in diverse animal phyla. There is also evidence that both connexins and innexins may, under some circumstances, expand their function from building intercellular channels to function as transmembrane (nonjunctional) pores. Innexins appear to form larger channels involving more subunits (16 innexins per gap junction channel versus 12 connexins), however, the topoogy of each subunit is similar in innexins and connexins. Four transmembrane domains anchor the protein in the membrane, extracellular loops are involved in docking and cytoplasmic domains contribute to regulation by factors such as protons, phosphorylation, and voltage. Both connexins and innexins display Vj‐dependent gating and selective formation of heterotypic channels, two properties related to the interesting phenomenon of voltage‐dependent rectification. For instance heterotypic channels formed by Cx26 and Cx32 rectify as do junctions resulting from heterotypic pairing of *Drosophila* innexins *ShakBL* and *ShakBN16*. The handful of structure‐function studies related to innexins so far suggest that like connexins, innexins require an amino terminus to form functional channels. As in connexins, the amino terminus plays a role in voltage‐gating. The first transmembrane domain (TM1) of connexins is more tightly packed than in innexins, however, mutations within TM1 can produce similar effects on function. For instance mutations within TM1 of both connexins and innexins can produce channels that are closed, but open in response to transjunctional voltage, a phenomenon thought to be related to partial collapse of the channel via disruption of interactions between helices. In addition, point mutations at some locations within the transmembrane domains of both connexins and innexins can induce calcium‐sensitive leak currents across the plasma membrane. These similarities are particularly interesting in light of the uncertain evolutionary origins of innexins and connexins.
